# Expression of concern: ‘TNF-α-induced NF-κB activation upregulates microRNA-150-3p and inhibits osteogenesis of mesenchymal stem cells by targeting β-catenin’ (2016), by Wang N *et al.*

**DOI:** 10.1098/rsob.230305

**Published:** 2023-08-31

**Authors:** Open Biology editorial team

Following the publication of ‘TNF-α-induced NF-κB activation upregulates microRNA-150-3p and inhibits osteogenesis of mesenchymal stem cells by targeting β-catenin’, the Editorial team have concerns regarding the validity of the data used in this study.

We are issuing an expression of concern here and investigating this as a matter of urgency; we will notify readers as to the results of our investigation as soon as possible.

